# Clinical observation of menopause hormone therapy in postmenopausal women with euthyroid and mild subclinical hypothyroidism

**DOI:** 10.1186/s12902-023-01269-7

**Published:** 2023-01-23

**Authors:** Wenxian Xu, Yizhou Huang, Linjuan Ma, Peiqiong Chen, Saisai Li, Ketan Chu, Yibing Lan, Chunming Li, Yang Song, Qian Ying, Jianhong Zhou

**Affiliations:** 1 Department of Gynecology Women’s Hospital School of Medicine, Zhejiang University, First Xueshi Rd, 310006 Hangzhou, People’s Republic of China; 2 Zhejiang Cancer Hospital, 310022 Hangzhou, People’s Republic of China

**Keywords:** Menopause hormone therapy, Postmenopausal women, Thyroid stimulating hormone, Mild subclinical hypothyroidism, Glucose and lipid metabolism

## Abstract

**Background:**

To evaluate the endocrine hormone and metabolic indices in postmenopausal women with euthyroid and mild subclinical hypothyroidism after menopause hormone therapy (MHT).

**Methods:**

A retrospective study of 587 postmenopausal women receiving MHT was conducted. Median (25–75th percentile) age was 52 (49–54) years. According to thyroid stimulating hormone (TSH) levels at initial diagnosis, the patients were divided into three groups: I (euthyroid with low normal TSH range, *n* = 460), II (euthyroid with upper normal TSH range, *n* = 106) and III (mild subclinical hypothyroidism, *n* = 21). After a continuous oral MHT regimen using the same estradiol potency for 6–18 month cycles, serum endocrine hormone and metabolic indices were reassessed.

**Results:**

Compared with baseline, serum TSH levels in groups I and II significantly changed but all values were within the normal range. No significant difference was observed in serum TSH levels in group III. After treatment, all serum free tri-iodothyronine and free thyroxine levels were within the normal range. Serum total cholesterol, triglyceride, fasting plasma glucose, fasting insulin levels and homeostasis model assessment of insulin resistance index had significantly decreased in group I. There were no significant differences in all observed lipid and glucose parameters in group III, before and after treatment.

**Conclusion:**

MHT did not affect thyroid function in postmenopausal women with euthyroid and mild subclinical hypothyroidism. MHT led to an improvement in lipid and glucose indicators in euthyroid women with low normal TSH range.

## Introduction

Menopause is characterized by the permanent cessation of menstrual periods as a consequence of a gradual and irreversible loss of ovarian function. The decline in ovarian function, metabolic changes and co-morbidities cause a wide spectrum of symptoms [1]. Menopause hormone therapy (MHT) has been extensive shown to relieve climacteric symptoms, including vasomotor symptoms and genitourinary syndrome [2]. Randomized trials also demonstrate positive effects on bone health and age-stratified analyses indicate more favorable effects in coronary heart disease and all-cause mortality in younger women (close proximity to menopause) compared with women at least a decade post onset of menopause [2].

Thyroid function and the gonadal axes are related throughout a woman’s fertility. The relationship between the two glands is mutual [3, 4]. Thyroid hormone increase the synthesis of sex hormone binding globulin (SHBG), testosterone, and androstenedione, reducing the clearance of estradiol and androgens while increasing the conversion of androgens to estrone [5]. The main role of estrogens in thyroid physiology is related to the increase in serum concentrations of thyroxine binding globulin (TBG) [6]. The fundamental essence of MHT is estradiol and it is important to note the effects of estrogens on thyroid function. The oral administration of estrogens causes a dose-dependent increase of the serum levels of TBG synthesized in the liver [7]. Previously, a study reported that oral MHT produced a marked increase in TBG levels and in total thyroxine (tT4); indeed, the average concentration of serum thyroid stimulating hormone (TSH) increased in postmenopausal women with primary hypothyroidism. In 40% of these women, the levothyroxine (L-T4) requirement also increased [8].

Many epidemiological surveys have reported that the prevalence of subclinical hypothyroidism is higher compared to overt hypothyroidism in elderly women [9, 10]. It is not uncommon for postmenopausal women who are seeking to start MHT to be affected by subclinical hypothyroidism. To date, few studies have assessed the effects of MHT on thyroid function in postmenopausal women with subclinical hypothyroidism, Furthermore, there is a sparsity of research exploring whether the use of MHT can lead to an increased risk of hypothyroidism or an increased demand for thyroxine.

Previous studies have demonstrated that postmenopausal women with hypothyroidism or subclinical hypothyroidism suffer adverse effects on lipid metabolism and consequent development of cardiovascular diseases [11]. Nevertheless, for postmenopausal women with thyroid dysfunction, it is unclear whether the use of MHT has favorable effects on lipid and glucose regulation.

This study aims to assess the endocrine hormone and metabolic status in postmenopausal women with euthyroid and mild subclinical hypothyroidism before and after MHT.

## Methods

### Study design and participants

A database was constructed for patients undergoing MHT between January 2010 to December 2020 at the menopause clinic in Women’s Hospital, Zhejiang University, School of Medicine, China. Baseline information, medical treatments, and follow-up records were collected and preserved in a self-developed MHT records management system. A total of 3108 MHT files had been established, from which we selected postmenopausal women, aged 40–60 years who had experienced cessation of menses over 12 months. Furthermore, these women had a serum FSH level over 40 IU/L and were within the normal range of fT3 and fT4, 0.3 ≤ TSH < 10 mIU/L at first visit. These women also received a continuous oral MHT regimen using the same potency of estradiol for 6—18 month cycles. Participants with a hysterectomy were given an estrogen-alone regimen. For participants with an intact uterus, estrogen was given combined with progesterone to provide endometrial protection. Progesterone additive regimens included sequential and continuous-combined treatment.

Participants were excluded if they reported: (i) a history of using estrogen and progestogen in the three months before first visit, (ii) a history of malignancies, (iii) induced menopause as a result of surgery, radiation or chemotherapy, (iv) diagnosis of any severe systematic or major organ diseases, (v) required treatment for thyroid dysfunction or a history of medication used to treat thyroid dysfunction, (vi) a history of psychological disorders and psychiatric issues, (vii) a history of autoimmune disorders, (viii) missing or incomplete blood results at first visit and follow-up, (ix) changed MHT regimen during treatment period. After screening, 2521 were excluded according to the inclusion and exclusion criteria, 587 postmenopausal women were ultimately enrolled in the present study.

Participants within the normal range of free tri-iodothyronine (fT3) and fT4 were divided into three groups according to the TSH values at the time of initial diagnosis. These were group I (euthyroid with low normal TSH range, 0.3 ≤ TSH ≤ 2.5 mIU/L, *n* = 460), group II (euthyroid with upper normal TSH range, 2.5 < TSH ≤ 4.5 mIU/L, *n* = 106) and group III (mild subclinical hypothyroidism, 4.5 < TSH < 10 mIU/L, *n* = 21). We aimed to group TSH levels and further stratify the differences in metabolic indicators under different TSH levels in the normal range, which had been performed previously [12–15]. The local ethical committee approved this study and an informed consent was obtained from each participant.

### Assessment and laboratory analyses

The assessments were carried out at baseline and after therapy. For women with multiple follow-ups during the treatment period, the last set of follow-up data were chosen to maximise follow-up durations. Age, time of amenorrhea, age of menarche, height, weight, systolic and diastolic blood pressure, circumferences of waist and hip were recorded at first visit. All women provided peripheral blood for measurement of thyroid hormone, reproductive endocrine hormones, and lipid and glucose metabolism parameters before and after MHT. Before blood sampling, participants were asked to fast for at least 8 h. The indices evaluated in this paper included serum levels of TSH, fT3, fT4, estradiol (E_2_), luteinizing hormone (LH), FSH, triglycerides (TG), total cholesterol (TC), high-density lipoprotein cholesterol (HDL-C), low-density lipoprotein cholesterol (LDL-C), fasting plasma glucose (FPG), and fasting insulin (FINS). In addition, a homeostasis model assessment of insulin resistance (HOMA-IR) was used to assess insulin resistance.

Serum TSH, fT3, fT4 and insulin levels were measured by chemiluminescent microparticle immunoassay (CMIA), using the associated commercially kids for Abbott Architect i2000 (Abbott Laboratories, Abbott Park, IL, USA). The intra- and inter-assay coefficients of variations (CVs) for TSH tests were less than 3% and 5%, respectively. Serum levels of FSH, LH, and E_2_ were determined by chemiluminescent immunoassay with commercially available kits, using an automated Roche Modular Analytics E170 immunoassay system (Roche Diagnostics, Meylan, France). Serum levels of TG, TC, HDL-C, and LDL-C were measured by standard enzymatic assays, while serum glucose was determined by hexokinase method, using the associated commercially kits for Beckman Coulter Chemistry Analyzer AU5800 (Beckman Coulter, Inc., Brea California, USA).

### Statistical methods

All statistical analyses were conducted using the Statistics Package for Social Sciences software, version 26.0 (SPSS Inc., Chicago, IL, USA). Data are reported as medians (25th percentile, 75th percentiles) for continuous variables and numbers (frequencies) for categorical variables, respectively. The Shapiro–Wilk test was utilized to analyze the normal distribution of continuous variables. For normally distributed data, inter-group comparisons were determined using the one-way ANOVA, and intra-group comparisons were evaluated using the paired-samples t test. For non-parametric distributed data, Wilcoxon signed-rank test was performed to assess significant differences within groups, and Kruskal–Wallis H test was chosen for comparison between independent groups. Additionally, categorical variables were compared using the Chi-square test or Fisher’s exact test, as appropriate. Results with *p* < 0.05 were considered statistically significant.

## Results

### The baseline characteristics of the participants

The present study included 587 postmenopausal women. The median (25—75th percentile) age of all participants was 52 (49—54) years. The median (25—75th percentile) menopause age of all participants was 48 (45—51) years. The baseline demographic characteristics of the women in the three groups were comparable. No significant differences were observed for age, age of amenorrhea, time since amenorrhea, age of menarche, residence, educational level, height, weight, body mass index (BMI) status, waist circumference, hip circumference, waist to hip ratio, or blood pressure readings across the three groups (Table 1).

**Table 1:** Characteristics of the participants at baseline

Variable	Group I (*n* = 460)	Group II (*n* = 106)	Group III (*n* = 21)	*H/χ*^*2*^	*p* value^a^
Age, y	52.00 (48.25, 54.00)	53.00 (49.75, 54.00)	53.00 (48.50, 55.00)	0.643	0.725
Age at menopause, y	48.00 (45.00, 51.00)	48.00 (45.00, 50.25)	49.00 (45.50, 52.00)	0.645	0.724
Time since amenorrhea, m	29.00 (17.00, 53.00)	32.50 (16.75, 64.25)	31.00 (18.00, 64.00)	1.597	0.450
Age of menarche, y	14.00 (14.00, 16.00)	14.00 (13.25, 15.00)	14.00 (13.00, 16.00)	3.199	0.202
Residence				3.070	0.523
Urban	381 (82.8)	89 (84.0)	15 (71.4)		
Suburb	50 (10.9)	11 (10.4)	3 (14.3)		
Rural	29 (6.3)	6 (5.7)	3 (14.3)		
Educational level				2.743	0.602
Less than or equal to middle school diploma or equivalent	144 (31.3)	29 (27.4)	6 (28.6)		
High school	172 (37.4)	35 (37.4)	8 (38.1)		
≥ College degree	144 (31.3)	42 (39.6)	7 (33.3)		
Height, m	1.60 (1.56, 1.63)	1.60 (1.58, 1.63)	1.61 (1.54, 1.64)	3.632	0.163
Weight, kg	56.00 (51.00, 60.00)	56.50 (53.00, 60.00)	58.00 (52.75, 61.13)	2.156	0.340
BMI, kg/m^2^	21.95 (20.34, 23.50)	21.76 (20.67, 23.75)	22.47 (20.37, 23.88)	0.795	0.672
Underweight	28 (6.1)	5 (4.7)	2 (9.5)	2.105	0.894
Normal weight	338 (73.5)	78 (73.6)	14 (66.7)		
Overweight	81 (17.6)	21 (19.8)	5 (23.8)		
Obesity	13 (2.8)	2 (1.9)	0 (0)		
Waist circumference, cm	76.00 (72.00, 82.00)	77.00 (72.00, 82.00)	78.50 (71.50, 85.50)	0.414	0.813
Hip circumference, cm	92.00 (89.00, 96.00)	93.00 (89.00, 96.00)	95.00 (88.50, 98.50)	1.353	0.508
Waist to hip ratio	0.83 (0.79, 0.87)	0.82 (0.78, 0.86)	0.83 (0.79, 0.87)	0.246	0.884
Hypertension				2.450	0.231
No	441 (95.9)	98 (92.5)	20 (95.2)		
Yes	19 (4.1)	8 (7.5)	1 (4.8)		
SBP, mmHg	120.00 (110.00, 124.00)	115.50 (110.00, 120.50)	120.00 (111.00, 120.00)	1.475	0.478
DBP, mmHg	76.00 (70.00, 80.00)	78.00 (70.00, 80.00)	75.00 (69.50, 80.75)	0.025	0.987

Data are expressed as numbers (frequencies) or medians (25th percentile, 75th percentiles) as appropriate

*p* < 0.05 was considered statistically significant

^a^Comparison between groups by Kruskal–Wallis H test for continuous variables or by χ^2^ test/Fisher’s exact test for categorical variables

*Abbreviation:* *BMI* body mass index, *SBP* systolic blood pressure, *DBP* diastolic blood pressure

### Endocrine indicators before and after MHT

For reproductive endocrine hormone indicators, no significant differences were observed in the baseline serum levels of FSH or LH between the three groups, while baseline serum level of E_2_ was shown a statistical difference, but all in a quite low level without clinical difference. The treatment of all postmenopausal women decreased the serum FSH level and increased the serum E_2_ level significantly in three groups. The serum LH level was decreased statistically but not in group III (Table 2).

**Table 2:** Thyroid and reproductive endocrine hormone parameters in euthyroid and subclinical hypothyroidism postmenopausal women, before and after MHT

Variable	Group I (*n* = 460)	Group II (*n* = 106)	Group III (*n* = 21)	*F/H*	*p* value^a^
TSH (mIU/L)					
Before MHT	1.46 (1.08, 1.88)	3.12 (2.73, 3.64)	5.14 (4.86, 6.09)	300.526	0.000
After MHT	1.75 (1.24, 2.21)	2.74 (2.29, 3.22)	5.17 (3.09, 6.19)	68.322	0.000
* t/Z*	5.050	2.559	0.847		
* p* value^b^	0.000	0.010	0.397		
fT3 (pmol/L)					
Before MHT	4.38 (3.96, 4.73)	4.45 (3.96, 4.87)	4.37 (4.13, 4.79)	1.276	0.528
After MHT	4.26 (3.91, 4.58)	4.19 (3.87, 4.66)	3.96 (3.47, 4.23)	2.683	0.070
* t/Z*	1.999	1.557	2.929		
* p* value^b^	0.047	0.119	0.013		
fT4 (pmol/L)					
Before MHT	13.82 (12.64, 15.04)	13.22 (12.38, 14.78)	13.33 (11.69, 14.74)	5.175	0.075
After MHT	13.36 (12.33, 14.58)	13.26 (12.32, 14.50)	12.83 (10.97, 14.13)	2.749	0.253
* t/Z*	3.181	1.153	0.853		
* p* value^b^	0.001	0.249	0.410		
LH(IU/L)					
Before MHT	36.42 (27.98, 46.59)	35.70 (27.98, 43.43)	40.27 (28.83, 43.54)	1.054	0.590
After MHT	31.75 (24.11, 42.51)	31.38 (22.57, 41.14)	29.71 (19.06, 38.40)	0.941	0.625
* t/Z*	6.157	2.252	1.614		
* p* value^b^	0.000	0.024	0.123		
FSH(IU/L)					
Before MHT	77.30 (63.89, 93.33)	75.08 (64.00, 90.96)	72.90 (54.75, 85.98)	2.341	0.310
After MHT	52.11 (39.51, 67.48)	51.61 (36.89, 67.47)	42.59 (34.16, 62.75)	2.002	0.368
* t/Z*	14.473	6.070	4.842		
* p* value^b^	0.000	0.000	0.000		
E_2_ (pmol/L)					
Before MHT	18.35 (18.35, 33.97)	18.40 (18.35, 38.05)	18.35 (18.35, 18.52)	7.828	0.020^c^
After MHT	71.41 (18.40, 170.30)	87.43 (25.51, 212.15)	79.38 (22.40, 193.30)	2.677	0.262
* t/Z*	10.970	6.059	3.294		
* p* value^b^	0.000	0.000	0.001		

Data are expressed as medians (25th percentile, 75th percentiles)

*p* < 0.05 was considered statistically significant

^a^*p* values are for between-group differences by Kruskal–Wallis H test/one way ANOVA

^b^*p* values are for within-group differences by Wilcoxon signed-rank test or paired-samples t test

^c^*p* < 0.05 and further between-group differences by post hoc test with Bonferroni correction as follows:

I vs. II *Z* = -26.895, *p* = 0.311; I vs. III *Z* = -72.162 *p* = 0.100; II vs. III *Z* = -99.057, *p* = 0.019

*Abbreviation:* *MHT* menopause hormone therapy, *TSH* thyroid stimulating hormone, *fT3* free tri-iodothyronine, *fT4* free thyroxine, *LH* luteinizing hormone, *FSH* follicle-stimulating hormone, *E*_*2*_, estradiol

For thyroid hormone indicators, no significant differences were observed between three groups in serum fT3 or fT4 before and after treatment. At follow-up, all serum fT3 and fT4 levels were in a normal range. In group I, patients increased the serum TSH level, decreased the serum fT3 and fT4 levels significantly. In group II, patients decreased the serum TSH level significantly with no differences in serum fT3 or fT4 levels. The serum TSH levels between two euthyroid groups still in a normal range. In group III, patients decreased the serum fT3 level significantly with no difference in serum TSH or fT4 levels (Table 2).

### Lipid and Glucose metabolism parameters before and after MHT

No statistically significant difference was observed between three groups with regard to baseline serum levels of TG, TC, HDL-C or LDL-C. At follow-up, postmenopausal women showed a marked decline in serum TG and TC levels significantly compared with baseline in group I and group II, while the serum levels of HDL-C or LDL-C had no significant changes. Moreover, no significant differences were noted in before-mentioned lipid parameters at follow-up and the changes from baseline in group III (Table 3).

**Table 3:** Lipid and glucose metabolism parameters in euthyroid and subclinical hypothyroidism postmenopausal women, before and after MHT

Variable	Group I (*n* = 460)	Group II (*n* = 106)	Group III (*n* = 21)	*F/H*	*p* value^a^
TG(mmol/L)					
Before MHT	1.07 (0.78, 1.51)	1.05 (0.87, 1.41)	0.84 (0.77, 1.90)	0.735	0.693
After MHT	1.00 (0.67, 1.36)	0.89 (0.75, 1.17)	1.02 (0.64, 1.44)	0.834	0.659
* t/Z*	2.860	2.657	0.632		
* p* value^b^	0.004	0.008	0.528		
TC(mmol/L)					
Before MHT	5.19 (4.61, 5.81)	5.27 (4.67, 5.84)	4.93 (4.32, 5.61)	1.484	0.476
After MHT	5.07 (4.44, 5.64)	4.76 (4.16, 5.35)	4.70 (4.18, 5.81)	5.332	0.070
* t/Z*	3.892	2.691	0.109		
* p* value^b^	0.000	0.007	0.913		
HDL-C(mmol/L)					
Before MHT	1.47 (1.26, 1.72)	1.44 (1.26, 1.72)	1.47 (1.15, 1.88)	0.141	0.932
After MHT	1.44 (1.25, 1.70)	1.46 (1.26, 1.58)	1.41 (1.24, 1.72)	0.150	0.928
* t/Z*	0.895	0.327	0.150		
* p* value^b^	0.371	0.745	0.883		
LDL-C(mmol/L)					
Before MHT	2.88 (2.44, 3.38)	2.91 (2.42, 3.62)	2.54 (1.94, 3.03)	4.685	0.096
After MHT	2.86 (2.41, 3.34)	2.59 (2.35, 3.15)	2.64 (2.16, 3.47)	4.034	0.133
* t/Z*	0.817	0.974	1.459		
* p* value^b^	0.414	0.330	0.145		
FPG (mmol/L)					
Before MHT	5.26 (4.97, 5.62)	5.26 (4.89, 5.56)	5.18 (4.93, 5.49)	1.175	0.556
After MHT	5.09 (4.85, 5.35)	5.12 (4.87, 5.36)	4.99 (4.57, 5.37)	1.669	0.434
* t/Z*	5.512	0.711	1.034		
* p* value^b^	0.000	0.477	0.301		
FINS (μU/ml)					
Before MHT	5.40 (4.10, 7.20)	5.60 (3.90, 8.00)	6.15 (3.70, 6.88)	0.086	0.958
After MHT	4.80 (3.60, 6.80)	5.10 (3.90, 6.90)	4.85 (3.85, 6.58)	0.874	0.646
* t/Z*	3.816	1.728	0.653		
* p* value^b^	0.000	0.084	0.513		
HOMA-IR					
Before MHT	1.33 (0.94, 1.77)	1.30 (0.88, 1.97)	1.38 (0.88, 1.60)	0.018	0.991
After MHT	1.08 (0.78, 1.55)	1.19 (0.86, 1.59)	1.13 (0.78, 1.52)	1.076	0.584
* t/Z*	4.442	1.806	0.992		
* p* value^b^	0.000	0.071	0.338		

Data are expressed as medians (25th percentile, 75th percentiles)

*p* < 0.05 was considered statistically significant

^a^*p* values are for between-group differences by Kruskal–Wallis H test/one way ANOVA

^b^*p* values are for within-group differences by Wilcoxon signed-rank test or paired-samples t test

Abbreviation: *MHT* menopause hormone therapy, *TG* triglyceride, *TC* total cholesterol, *HDL-C* high-density lipoprotein cholesterol, *LDL-C* Low-density lipoprotein cholesterol, *FPG* fasting plasma glucose, *FINS* fasting insulin, *HOMA-IR* homeostasis model assessment of insulin resistance

No observable difference was demonstrated in terms of the baseline serum levels of FPG, FINS or HOMA-IR in three groups. As compared to baseline values, group I postmenopausal women exhibited a significant decline in serum levels of FPG and FINS, as well as HOMA-IR after MHT. In group II and III women, the serum concentrations of FPG, FINS and HOMA-IR were not affected significantly compared with baseline (Table 3).

## Discussion

This retrospective study aimed to evaluate euthyroid and mild subclinical hypothyroidism postmenopausal women who underwent MHT, comparing endocrine hormone levels and metabolic status before and after MHT. In this study, there were no statistically significant differences in age, time of menopause, BMI or other baseline indices among the three groups before medication. All participants received oral MHT with same potency of estrogen, and the medication regimens among the three groups were comparable.

Consensus to date suggests that MHT through exogenous supplementation of estrogen can be given to reduce the level of FSH in serum, thus maintaining a certain E_2_ level that meets the needs of various organs in the body, and consequently relieves menopausal symptoms [16]. In our study, the serum FSH decreased and E_2_ increased in all three groups compared with the baseline, which is consistent with previous studies. Meanwhile, no difference was observed at follow-up between the three groups.

Previous studies have examined the effects of MHT on thyroid function in euthyroid postmenopausal women. In the study by Benencia et al. [17], postmenopausal women who used oral MHT resulted in an increase in serum TBG and tT4 levels at 3, 6 and 12 months, but within the normal range. Moreover, serum TSH, fT4, and total tri-iodothyronine (tT3) levels did not change significantly and remained within the normal range. Ceresini et al. [18] reported that serum TSH did not change significantly after 1 year of estrogen therapy in euthyroid postmenopausal women. Marqusee et al. [19] demonstrated that the administration of conjugated estrogen for 6 weeks increased serum TSH concentrations, but did not alter fT4 index values in postmenopausal women. Indeed, serum TSH values remained within the normal range. For postmenopausal women with thyroid dysfunction, one small study explored the effects caused by MHT. In a 48-week study, Arafah investigated the effects of oral MHT on thyroid function in postmenopausal women and in women with normal thyroid function [8, 20]. TBG and tT4 levels increased, while fT4 and TSH levels did not change. In women with primary hypothyroidism who received L-T4 therapy, oral MHT produced a marked and sustained increase in TBG levels and a parallel increase in tT4 levels. However, in contrast to euthyroid women, the serum fT4 levels decreased significantly and the average serum TSH levels increased markedly, while 40% patients had their L-T4 doses increased after their TSH levels had exceeded the predetermined limits set in the protocol.

Our study found that after MHT, postmenopausal women with euthyroid remained in the normal range of serum TSH, fT3 and fT4 levels, this result is consistent with previous researches. In our study, postmenopausal women with mild subclinical hypothyroidism had significantly decreased serum fT3 levels, while still within the normal range after MHT. Meanwhile, serum TSH and fT4 showed no significant change before and after MHT. Our study is the first to analyze the effects of MHT on mild subclinical hypothyroidism in postmenopausal women. Our findings suggested that MHT does not have a significant effect on thyroid function, and does not lead to increased demand for thyroxine. Furthermore, MHT does not aggravate the trend of clinical hypothyroidism for mild subclinical hypothyroidism in postmenopausal women.

Consistent results from several large randomized controlled trials indicate that MHT regimens produced a significant increase in HDL-C and TG levels, but with reductions in LDL-C levels, which also led to a decline in FPG levels and a decreased likelihood of type 2 diabetes [21–24]. Our study demonstrated that MHT led to improvements in lipid parameters in euthyroid postmenopausal women, which is consistent with previous studies assessing lipid indicators. We observed no significant changes in lipid parameters in postmenopausal women with mild subclinical hypothyroidism, before and after treatment. It is possible that elevated TSH impairs the ability of MHT to improve lipid levels but further research is required to confirm this. In this study, we found that MHT significantly decreased FPG, FINS, and HOMA-IR in euthyroid postmenopausal women with low normal TSH range. However, there were no significant differences between euthyroid women with upper normal TSH level or women with mild subclinical hypothyroidism. Based on these results, women with low normal TSH range showed more obvious within-group improvements in glucose and lipid metabolism after MHT.

There are several potential limitations to this study. First, the investigation was a retrospective analysis, and this study did not include a placebo group or a control group that did not receive MHT. Second, the sample size of women in group III was too small, which limits statistical inference to a larger dataset or stratifying the analysis on the type of MHT received (estrogen-alone versus estrogen combined with progesterone). Finally, selection and recall bias existed in the recruitment of the participants. Given these limitations, further prospective studies with larger sample sizes and homogeneous hormone therapies are required.

## Conclusions

MHT can effectively regulate reproductive hormone levels and does not affect thyroid function in euthyroid and mild subclinical hypothyroidism postmenopausal women. MHT led to significant improvements in lipid and glucose levels in euthyroid postmenopausal women with low normal TSH range, while there was no significant difference in lipid or glucose indicators in postmenopausal women with mild subclinical hypothyroidism.

## Data Availability

The datasets used and/or analyzed during the current study are available from the corresponding author on reasonable request.
