# Correction: clinical observation of menopause hormone therapy in postmenopausal women with euthyroid and mild subclinical hypothyroidism

**DOI:** 10.1186/s12902-023-01312-7

**Published:** 2023-03-07

**Authors:** Wenxian Xu, Yizhou Huang, Linjuan Ma, Peiqiong Chen, Saisai Li, Ketan Chu, Yibing Lan, Chunming Li, Yang Song, Qian Ying, Jianhong Zhou

**Affiliations:** 1 Department of Gynecology Women’s Hospital School of Medicine, Zhejiang University, First Xueshi Rd, 310006 Hangzhou, People’s Republic of China; 2 Zhejiang Cancer Hospital, 310022 Hangzhou, People’s Republic of China

**Correction to:** ***BMC Endocr Disord*23, 21 (2023).**

10.1186/s12902-023-01269-7

Following publication of the original article [1], the authors reported that affiliation 2 was mistakenly assigned to Jianhong Zhou.

The correct affiliation for Jianhong Zhou is only affiliation 1: Department of Gynecology Women’s Hospital School of Medicine, Zhejiang University, First Xueshi Rd, 310006 Hangzhou, People’s Republic of China.

The original article [1] has been updated.

## References

[1] Xu W, Huang Y, Ma L (2023). Clinical observation of menopause hormone therapy in postmenopausal women with euthyroid and mild subclinical hypothyroidism. BMC Endocr Disord.

